# Humidifier Use and Prone Positioning in a Patient with Severe COVID-19 Pneumonia and Endotracheal Tube Impaction Due to Highly Viscous Sputum

**DOI:** 10.7759/cureus.8626

**Published:** 2020-06-15

**Authors:** Ryu Sugimoto, Tsuneaki Kenzaka, Moemi Fujikawa, Satoru Kawasaki, Hogara Nishisaki

**Affiliations:** 1 Department of Internal Medicine, Hyogo Prefectural Tamba Medical Center, Tanba, JPN; 2 Division of Community Medicine and Career Development, Kobe University Graduate School of Medicine, Kobe, JPN

**Keywords:** coronavirus 2019 (covid-19), sars cov-2, sars-cov-2 (severe acute respiratory syndrome coronavirus -2), covid-19 respiratory failure high altitude pulmonary edema high flow nasal cannula acute respiratory distress syndrome, invasive mechanical ventilation, tube impaction, key words corona, coronavirus disease 2019

## Abstract

COVID-19 can lead to severe pneumonia, requiring mechanical ventilation. While increased sputum secretion could cause airway obstruction during mechanical ventilation, there are few reported cases in the literature. We report a case of a 65-year-old man with diabetes and severe COVID-19 pneumonia requiring mechanical ventilation and treated with hydroxychloroquine, azithromycin, nafamostat, and prone positioning. Initially, mechanical ventilation consisted of a heat moisture exchanger, endotracheal tube aspiration, and subglottic secretion drainage using a closed suction system. However, endotracheal tube impaction by highly viscous sputum occurred during this mechanical ventilation system. Replacing the endotracheal tube, the use of a humidifier instead of a heat moisture exchanger, and prone positioning contributed to the patient being weaned off mechanical ventilation. Although anti-aerosol measures are important for severe COVID-19 pneumonia, attention should be given to potential endotracheal tube impaction during mechanical ventilation.

## Introduction

The SARS-CoV-2 infection (hereinafter, COVID-19), which first occurred in China in December 2019, has now spread worldwide. While around 80% of COVID-19 cases recover after having mild symptoms, around 20% of cases become severe symptoms and around 3% of patients require mechanical ventilation [1-2]. In addition, cases of acute respiratory distress syndrome (ARDS) tend to have serious outcomes, where around 50% of such cases have resulted in death [3]. The Japan Society of Intensive Care Medicine and Japanese Society of Respiratory Care Medicine have mentioned that airway impaction from increased sputum secretion is characteristic in patients with severe COVID-19 on mechanical ventilation, which is an issue to which healthcare workers should pay attention [4]. However, There have been almost no detailed case presentations thus far.

Herein, we describe a patient with severe COVID-19 pneumonia for whom mechanical ventilation was complicated due to endotracheal tube impaction from sputum. The patient and his family members were informed about this report and consented to publication. The use of each therapeutic drug had been approved by the hospital’s ethics committee in advance.

## Case presentation

A 65-year-old Japanese man was transported urgently to our hospital with the chief complaint of difficulty in breathing. The patient had a history of hypertension and type 2 diabetes, for which he was receiving treatment. The patient was a non-smoker. The patient was tested for SARS-CoV-2 by reverse transcription-polymerase chain reaction (RT-PCR; hereinafter, PCR test) 12 days earlier because of his history of close contact with someone else in his family who had contracted COVID-19, but his result was negative at that time. The patient had fever (around 38℃), general malaise, and difficulty in breathing seven days before the hospital visit. After deciding to monitor the condition from home, the symptoms seemed to be improving, but the breathing worsened the day before the hospital visit. The PCR test was conducted again on the day of the hospital visit, and the patient tested positive for the virus on the same day. As the patient’s peripheral capillary oxygen saturation (SpO_2^)^ _was around 80% on room air, he was urgently transported to the hospital.

The physical findings were as follows: height, 175 cm; body weight, 92 kg; body mass index, 30.0 kg/m^2^; clear consciousness; body temperature, 38.2℃; blood pressure, 144/79 mmHg; heart rate, 96 bpm; regular respiratory rate, 20 breaths per min; SpO_2_, 96% (reservoir mask: O_2_ 8 L/min). The heart sound was regular, with no murmurs. Crackling sounds were heard from the dorsal side of the lower lobes of both lungs.

Hematological findings are shown in Table 1.

**Table 1:** Laboratory data upon admission HbA1c: glycated hemoglobin

Parameter	Recorded value	Standard value
White blood cell count	11,260/µL	4500–7500/µL
Neutrophils	90.7%	42–74%
Lymphocytes	5.3%	18–50%
Monocytes	4.0%	1–10%
Hemoglobin	13.2 g/dL	11.3–15.2 g/dL
Platelet count	147 × 10^3^/µL	130–350 × 10^3^/µL
Prothrombin time / International normalized ratio	1.03	0.80–1.20
Activated partial thromboplastin time	32.9 s	26.9–38.1 s
D-dimer	1.7 μg/mL	<1.0 μg/mL
C-reactive protein	11.3 mg/L	≤0.60 mg/dL
Procalcitonin	0.97 ng/mL	≤0.05 ng/mL
Total protein	7.3 g/dL	6.9–8.4 g/dL
Albumin	3.6 g/dL	3.9–5.1 g/dL
Total bilirubin	0.8 mg/dL	0.2–1.2 mg/dL
Aspartate aminotransferase	61 U/L	11–30 U/L
Alanine aminotransferase	40 U/L	4–30 U/L
Lactase dehydrogenase	467 U/L	109–216 U/L
Creatine kinase	956 U/L	40–150 U/L
Blood urea nitrogen	34.3 mg/dL	8–20 mg/dL
Creatinine	1.68 mg/dL	0.63–1.03 mg/dL
Sodium	137 mEq/L	136–148 mEq/L
Potassium	4.2 mEq/L	3.6–5.0 mEq/L
Chloride	102 mEq/L	98-108 mEq/L
Glucose	157 mg/dL	70–109 mg/dL
HbA1c	8.9%	5.6–5.9%
Ferritin	812 ng/mL	20–250 ng/mL
Troponin I	114.3 pg/mL	≤26.2 pg/mL
Brain natriuretic peptide	87.7 pg/mL	≤18.4pg/mL

There was neutrophil-dominated elevation in white blood cells, as well as elevated C-reactive protein (CRP). We observed renal dysfunction, as well as elevated levels of liver enzymes and creatine kinase. There was a mild increase in the levels of ferritin and procalcitonin. Urinalysis showed urine protein (2+), urine sugar (4+), and urine occult blood (2+). The urine β2MG level was 1,596 µg/L. Urinary pneumococcal and Legionella antigen tests were both negative.

**Figure 1:**
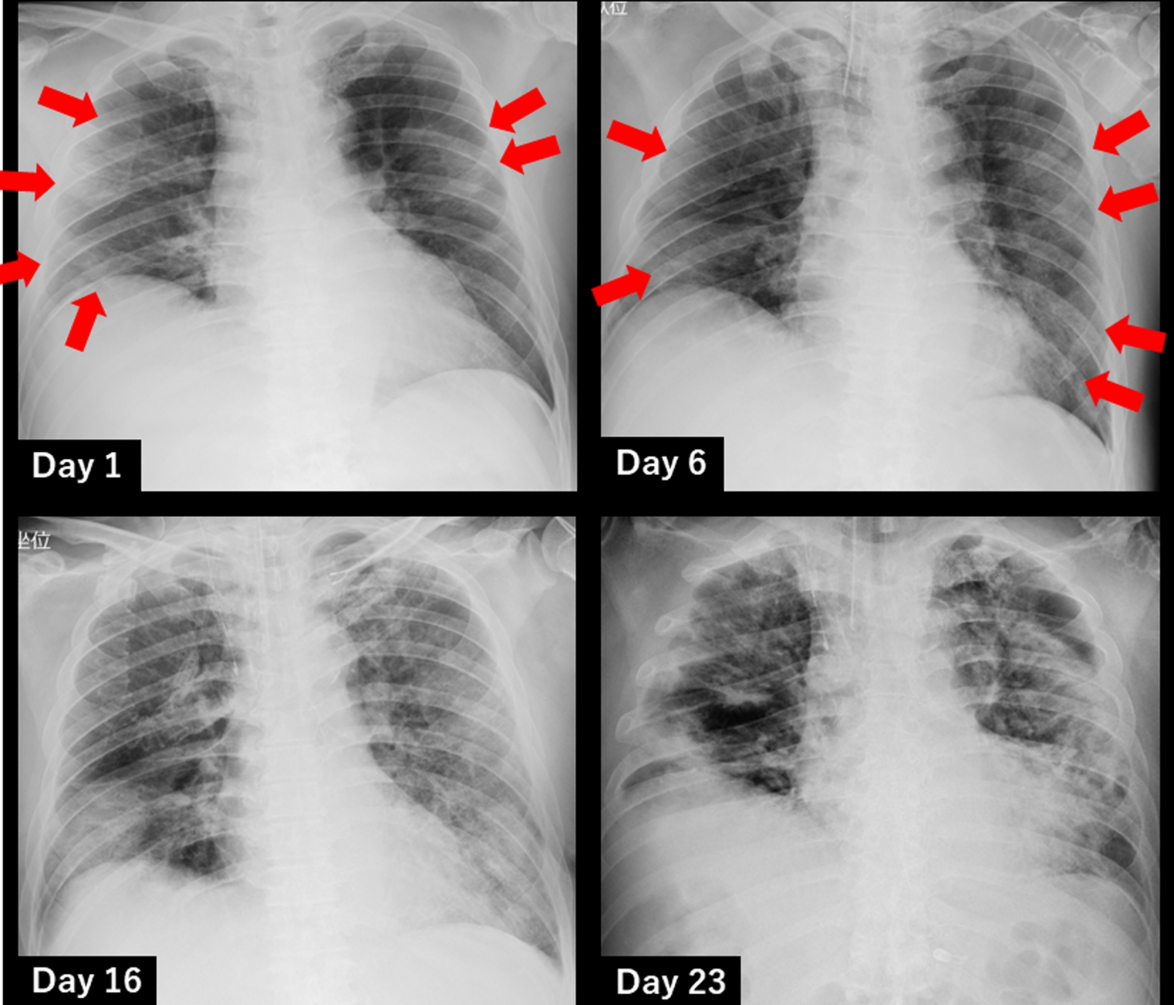
Changes in chest X-ray images

Chest X-ray images (Figure 1) showed ground-glass opacities and infiltrative shadows in both lungs.

**Figure 2:**
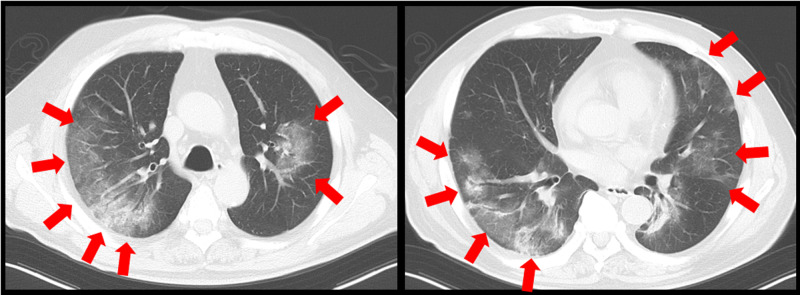
Chest computed tomography scans taken at admission

Plain chest computed tomography (CT) scans (Figure 2) showed non-regional ground-glass opacities in both lungs and some infiltrative shadows.

**Figure 3:**
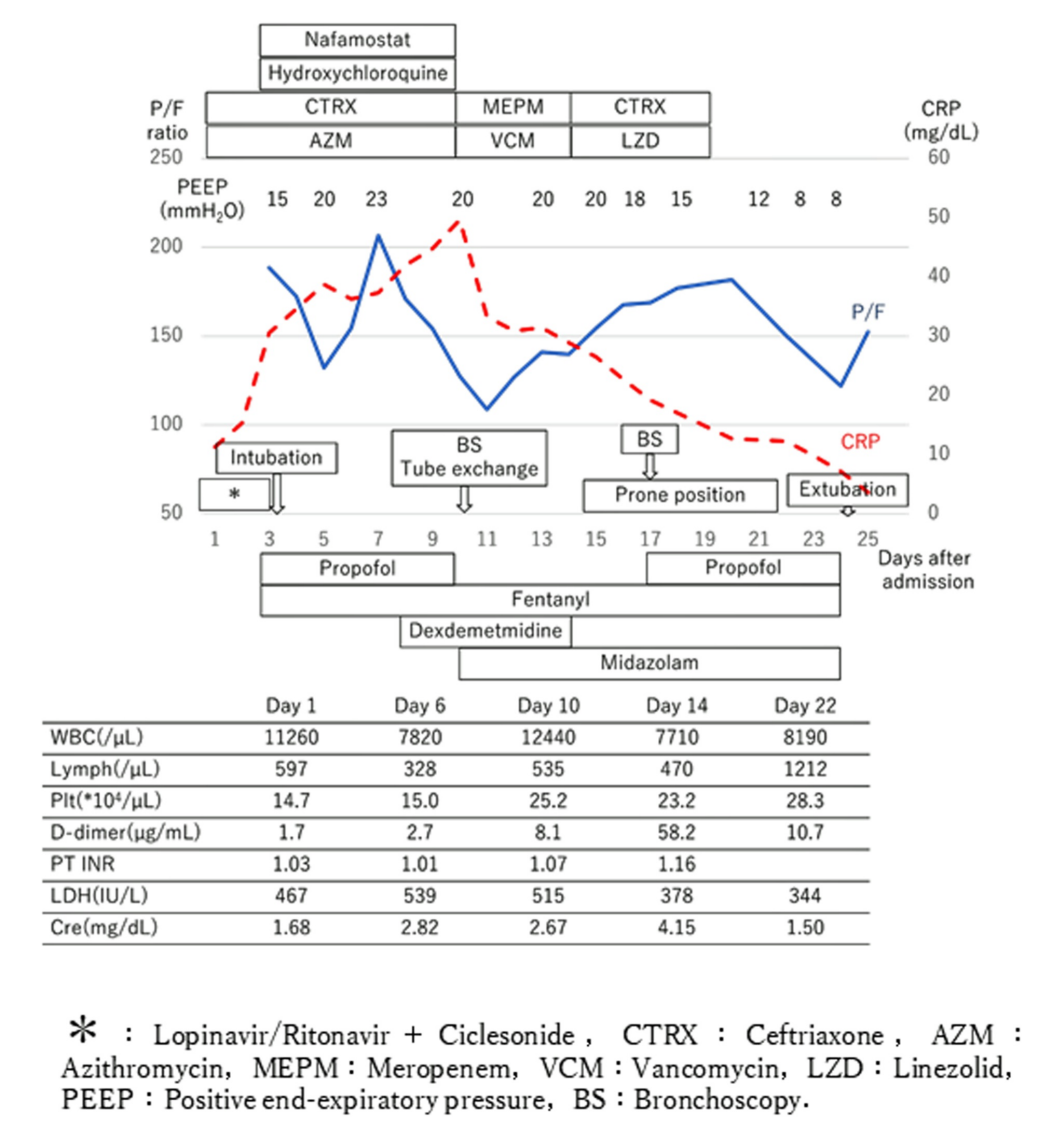
Chart showing the clinical course after admission

The patient was transferred to a negative-pressure room in the intensive care unit for the management of COVID-19 pneumonia. Figure 3 shows the patient’s clinical course after admission.

**Figure 4:**
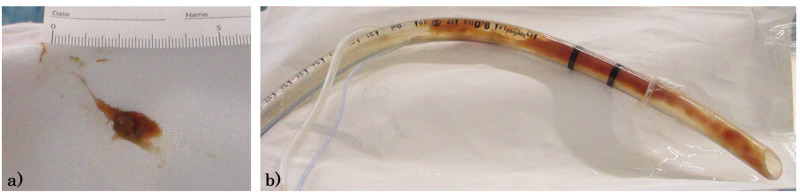
Sputum images: a) aspirated sputum, b) sputum in the intubation tube

From Day 1 of admission, the patient was started on treatment with oral lopinavir/ritonavir and inhaled ciclesonide. In addition, the patient was given an intravenous infusion of ceftriaxone and oral azithromycin treatments in consideration of complications with bacterial pneumonia. However, there was little improvement in his respiratory condition, and the patient remained in a condition that required high flow oxygen. For this reason, the patient was intubated on Day 3 after admission by rapid sequence induction using rocuronium to begin management under mechanical ventilation. As part of mechanical ventilation, a heat moisture exchanger was used as an anti-aerosol measure. At the same time, a closed suction system was used for endotracheal tube aspiration and subglottic secretion drainage. Inhaled ciclesonide and lopinavir/ritonavir treatments were discontinued at the start of mechanical ventilation and, instead, the patient was started on 400 mg/day oral hydroxychloroquine, 250 mg/day oral azithromycin, and 200 mg/day continuous intravenous infusion of nafamostat. Mechanical ventilation and fluid volume control were performed according to the ARDS protocol, with a tidal volume of 6 mL/kg (ideal body weight), plateau pressure <30 mmH_2_O, and restricted infusion volume, but it was difficult to maintain oxygenation and ventilation without using a high airway pressure with a plateau pressure ≧25 mmH_2_O. By Day 10, the patient developed ventilator-associated pneumonia and was started on meropenem and vancomycin treatments. In the afternoon of the same day, the tidal volume dropped rapidly to around 100-150 mL, and the patient became tachypneic with a respiratory rate exceeding 30 breaths per min. As the EtCO_2_ increased to around 120 mmHg and ventilation could not be maintained even with a driving pressure of 20 mmH_2_O, it was decided that an emergency bronchoscopy would be performed. The administration of a muscle relaxant to perform bronchoscopy also decreased the patient’s SpO_2_. The patient’s SpO_2_ was 92% even at FiO_2_ 1.0. When the bronchoscope was inserted after stopping the ventilator to avoid aerosol exposure, we found that the endotracheal tube was full of reddish-brown sputum, almost completely impacting the tube lumen. Aspiration was not possible even when the suction pressure was increased, and the SpO_2_ and tidal volume remained below 90% and 100 mL, respectively, even at a positive end-expiratory pressure (PEEP) of 20 mmH_2_O and driving pressure of 30 mmH_2_O. For this reason, we pushed the adhered matter into the trachea and removed the bronchoscope while aspirating again, and we managed to aspirate the Azuki bean-sized, viscous, and soft reddish-brown sputum. Both the SpO_2_ and the tidal volume improved after this. When the bronchoscope was re-inserted, similar sputum was found adhered to the end of the endotracheal tube. As the patient’s SpO_2 _level remained stable, a tube exchanger was used to replace the endotracheal tube. The lumen of the removed endotracheal tube was covered with highly viscous reddish-brown sputum (Figure 4).

Both oxygenation and tidal volume improved after tube replacement, and from this point on, the use of the heat moisture exchanger was discontinued and replaced by a humidifier. Taking note of the previous reports on the drug administration period where an antiviral effect is anticipated, the use of hydroxychloroquine and nafamostat ended on Day 11. However, as the patient continued to show little improvement in his respiratory condition, the patient started to undergo 5-6 h/day of prone positioning from Day 15. When bronchoscopy was performed again on Day 17, we found no sputum adhering to the inside of the endotracheal tube. As shown by the patient’s P/F ratio (PaO_2_/FiO_2_) in Figure 3, his respiratory condition improved gradually after the induction of prone positioning. Antimicrobial treatments ended by Day 19, while prone positioning was completed by Day 21. From Day 22, we started to interrupt the patient’s sedation once a day to check his consciousness (Spontaneous Awakening Trial; SAT) and to see whether he could be extubated for unassisted breathing (Spontaneous Breathing Trial; SBT). The patient performed SBT successfully from the same day it was started and was thus extubated on Day 24. To minimize the healthcare staff’s exposure to aerosol during extubation, rather than performing a cuff leak test, the patient was extubated with a large plastic bag covering his head, and a surgical mask was put on him after extubation. Although a high-flow nasal cannula was used immediately after extubation, by the next day, there were no issues with the patient’s respiratory condition even with a 4 L/min face mask. By Day 37, the patient’s respiratory condition stabilized and oxygen administration was discontinued. The patient was transferred to another hospital for the rehabilitation of his swallowing and limb functions on Day 45.

## Discussion

We encountered a case of a patient with COVID-19 for whom the continuation of mechanical ventilation proved difficult after endotracheal tube impaction with highly viscous sputum. Endotracheal tube replacement followed by humidifier use and prone positioning contributed to the patient being weaned off mechanical ventilation.

The Japan Society of Intensive Care Medicine and Japanese Society of Respiratory Care Medicine have mentioned that airway impaction from increased sputum secretion is characteristic in patients with severe COVID-19 on mechanical ventilation, which is an issue that healthcare workers should be aware of [4]. Our patient had an impacted endotracheal tube due to the accumulation of highly viscous reddish-brown sputum. The reddish-brown color was indicative of high levels of red blood cell components. The suction tube could be inserted into the endotracheal tube, and there was a Bougie effect in the tube, but the sputum was too viscous to remove by aspiration. As such, we responded by replacing the tube. The main mechanism in the pathophysiology of rapidly deteriorating COVID-19 patients is thought to be due to ARDS caused by excessive inflammation [5]. Many studies have suggested that COVID-19 patients undergo rapid deterioration through an immunological mechanism known as a “cytokine storm,” wherein the release of inflammatory markers causes a positive feedback loop that brings about ARDS, multiple organ failure, and death [6]. The virus is thought to proliferate in alveolar epithelial cells [7], induce local inflammation, and cause lung damage while exhibiting an overactivated immune response by pathogenic Th1 cells and inflammatory monocytes [8]. As the patient showed a rapid elevation in D-dimer levels from several days before Day 10 when he exhibited endotracheal tube impaction, we surmise that he may have experienced coagulation abnormalities and inflammation of blood vessels around the lungs, resulting in the generation of highly viscous sputum rich in red blood cell component content. Furthermore, although the use of a heat moisture exchanger is recommended for mechanical ventilation in severe COVID-19 pneumonia patients [9], we did not observe any viscous sputum during the bronchoscopy performed after switching from a heat moisture exchanger to a humidifier. Although the patient’s condition showed improvements after replacing the endotracheal tube, the heat moisture exchanger may not have provided adequate humidification, and a drier management approach taken by restricted infusion volume may have promoted airway impaction with viscous sputum.

Treatment for COVID-19 consists primarily of supportive care by means of oxygen administration and mechanical ventilation, among other means. The recommendation is to focus on “lung-protective strategies” based on the ARDSnet study [10] in which the mortality rate in ARDS cases of COVID-19 patients improved with a low ventilation volume [5]. The tidal volume is first set to 6 mL/kg based on the ideal body weight. If the patient develops lung damage and progresses to ARDS, the lungs gradually collapse and form a shunt, reducing functional lung capacity. The low-volume ventilation strategy reduces the extent by which functional lung capacity decreases. It has been suggested that prone positioning is effective for patients with severe COVID-19 pneumonia that have progressed to ARDS [11]. It is recommended that patients are kept for 12 to 16 hours per day in the prone position and that a change in position is carried out when there are enough medical staff around such as during work shifts. During the change in body position, it is necessary to try and avoid the interruption of mechanical ventilation as much as possible. Our hospital lacks experience in prone positioning for ARDS in general, including diseases other than COVID-19, and for this reason, we were not using prone positioning at first. However, as the patient failed to show an improvement in his respiratory condition even after we had addressed the endotracheal tube impaction, we started him on prone positioning therapy with the aim of improving lung compliance and the ventilation/perfusion ratio imbalance. Prone positioning was induced for five to six hours per day during the day to ensure that there were enough medical staff around and that we would be able to troubleshoot in the event of a problem. Prone positioning proved effective for this case, as the patient’s respiratory conditions gradually improved after the start of treatment.

As of April 28, 2020, there is no antiviral treatment that has been proven effective in humans to combat COVID-19. All medications administered to this patient were those that could be obtained at our hospital. We began treating the patient using lopinavir/ritonavir and ciclesonide, but as the lopinavir/ritonavir formulation used in this hospital has been shown to have decreased blood concentration through gastric tube administration and was not effective when used in randomized controlled trials [12], its use was discontinued. Ciclesonide was also discontinued, as we did not expect that enough of the drug would reach the alveolar epithelium while the patient was under mechanical ventilation. In this hospital, we decided to use hydroxychloroquine, azithromycin, and nafamostat, as it is expected that hydroxychloroquine provides an antiviral effect and suppresses the production of inflammatory cytokines [13]. Combined hydroxychloroquine/azithromycin treatments have proven effective and safe in a small-scale non-randomized study [14], and it is anticipated that nafamostat controls the early stages of a COVID-19 infection and suppresses the IL-6 amplification circuit [8]. Even though it is not clear how effective the drugs were for this case, it is expected that the results of future clinical trials will reveal more information.

## Conclusions

Patients with severe COVID-19 pneumonia can experience an impacted airway from increased sputum excretion, and our patient experienced a deterioration in his respiratory condition after an almost complete impaction of the inside of the inserted endotracheal tube. The use of a humidifier may be effective if a patient exhibits viscous sputum. Although only applied for short periods of time, prone positioning proved effective in improving the respiratory conditions in our patient. Although anti-aerosol measures are important for severe COVID-19 pneumonia, it is necessary to pay attention to tube troubles caused by the viscous sputum generated while a patient is being managed using a heat moisture exchanger.
